# Can Active Video Games Improve Physical Activity in Adolescents? A Review of RCT

**DOI:** 10.3390/ijerph17020669

**Published:** 2020-01-20

**Authors:** Wanda M. Williams, Cynthia G. Ayres

**Affiliations:** School of Nursing, Rutgers University, the State University of New Jersey, Camden, NJ 08102-1530, USA; cgayres@rutgers.edu

**Keywords:** physical activity, adolescents, teens, youth, gamification, exergaming, active video games

## Abstract

Children and adolescents are not meeting the required federal physical activity (PA) guidelines established by the United States Department of Health and Human Services. It is critical that a regular pattern of PA is established in their youth to ensure ongoing PA into adulthood. However, many programs implemented during adolescence have shown limited effects, stressing the need for more innovative approaches to generate more interest and maintenance of PA behavior in this age group. Active video games (AVGs) or exergaming may prove to be an innovate process to improve PA in children and adolescents. A literature review was conducted to explore if active video games or exergaming could be an effective intervention to improve physical activity in adolescents. Active video games, also called “exergames”, are a category of video games that require movement or physical exertion to play the game. The methodology consisted of searching Academic Search Premier, CINAHL, The Cochrane Library, ERIC, PubMed, and Web of Science databases. Inclusion criteria involved only youth aged 12 to 19 years, randomized controlled trials (RCTs), and studies within the last 12 years. The following search terms were used: exergaming or active video games; physical activity or exercise; adolescents or youth; RCT or randomized clinical trial. The outcome indicates that exergaming or active video games can be an effective tool to improve PA in adolescents that will be more acceptable and sustainable than many conventional approaches.

## 1. Introduction

Being physically inactive or failure to meet the daily physical activity (PA) recommendations is considered to be one of the leading risk factors for many chronic diseases (e.g., heart disease, stroke, cancer, diabetes, and chronic respiratory diseases) [1]. The impact of physical inactivity on our society can be seen in shorter life expectancy, poorer health outcomes, and decreased quality of life [2]. Research shows that regular physical activity is essential and beneficial to improving muscular and cardiorespiratory fitness; improving bone and functional health; and reducing the risk of many chronic diseases and some forms of cancer [3,4]. Yet, children of all ages are failing to meet the required level of physical activity. Physical activity is defined as any bodily movement produced by skeletal muscles that requires energy expenditure [5]. 

The Physical Activity Guidelines for Americans “recommend that children and adolescents ages 6 through 17 years should do 60 minutes (1 hour) or more of moderate-to-vigorous physical activity daily” [6]. The guidelines outline that most of the 60-minute activity “should be either moderate- or vigorous-intensity aerobic physical activity and should include vigorous-intensity physical activity on at least 3 days a week; muscle-strengthening should also be a key part of these activities, as well bone-strengthening activities on at least 3 days a week” [6]. Unfortunately, self-reported data of high school students in the United States indicate that the prevalence of adolescents meeting the recommendation of ≥60 minutes of physical activity on all seven days of the week was 27.1% nationwide and declined from the 9th (31.0%) to 12th (23.5%) grades [7,8]. 

Physical activity has been a leading health indicator for the Healthy People (HP) program since the program’s conception in 1979 [9,10]. As we move into HP 2030, physical activity continues to be a leading health indicator due to the key role that it plays in disease prevention and promotion of lifelong health [3,4]. Therefore, effective and sustainable interventions are needed, especially for adolescents. Adolescence is a major transition period from childhood to adulthood. Many physical changes occur during this time period. Maintaining adequate physical activity habits could significantly translate into better health habits in adulthood [6]. 

One solution to increasing physical activity in adolescents may involve the use of active video games. Active video games (AVGs), also called “exergames”, are a category of video games that require movement or physical exertion to play the game [8]. Today’s youth are living in the age of technology, where active video games may be the ideal solution to promote and encourage more energy expenditure during their free time.

Therefore, the purpose of this review is to synthesize the current evidence based on randomized controlled trials (RCTs) regarding the effects of exergaming or AVGs on improving or increasing physical activity among adolescents (ages 12 to 19 years—this age range is usually the period identified as adolescence). The question used as the search strategy to guide this review was, “In the adolescent population, does the use of video gaming improve physical behavior?” 

## 2. Methods

### 2.1. Data Sources and Searches

A comprehensive electronic search of the following databases was conducted: Academic Search Premier, CINAHL, The Cochrane Library, ERIC, PubMed, and Web of Science. The articles published in English between 2007 and 2019 were searched and obtained using the following key terms: randomized control trials or RCT, physical activity or exercise, adolescents, youth, active video games, and exergaming. These keywords were searched separately on each database. Additional searches were also done from the reference lists of some selected articles and included in the review. 

### 2.2. Inclusion and Exclusion Criteria

Prior to inclusion, titles and abstracts of the searched articles were screened for relevance prior to being included in the review. Next, full texts of the articles were obtained and reviewed based on the inclusion criteria. Inclusion criteria included: (1) randomized controlled trial as the study design, (2) the population studied comprised individuals between 12 and 19 years of age, (3) intervention included active video games (AVGs) or exergaming with one outcome directed at increased physical activity, and (4) published in a peer-reviewed journal between 2007 and 2019. Studies were excluded if they were published in languages other than English. 

### 2.3. Data Extraction

This review was conducted and reported in accordance with the guidelines outlined in the Preferred Reporting Items for Systematic Reviews and Meta-Analyses (PRISMA) statement [11]. The articles selected for this review are summarized and presented in Table 1: author, year published, framework/theory, randomization process, dose/duration, sample, gaming process/system, physical activity measurement, and results/findings. 

### 2.4. Risk of Bias Assessment

The Cochrane Collaboration Risk of Bias tool was used to assess for bias [12]. Two reviewers independently assessed the methodological quality of each article that was included in this review and resolved any lack of consensus by discussion. The methodological criteria were closely followed.

## 3. Results

### 3.1. Search Results and Article Selection

**Figure 1:**
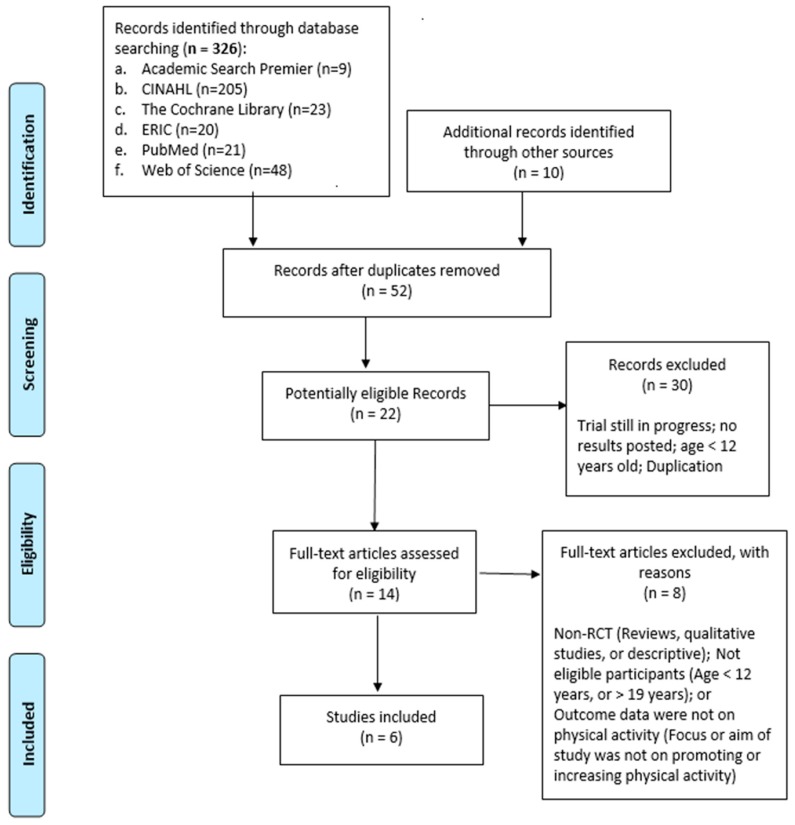
Preferred Reporting Items for Systematic Reviews and Meta-Analyses (PRISMA) http://www.prisma-statement.org.

Article selection was done according to the PRISMA guidelines. The comprehensive steps of the selection process and the number of articles in each step can be seen in Figure 1. Studies were excluded if the participants’ age was less than 12 or more than 19 years of age; if they were not a randomized controlled trial; or if the primary outcome was not directed at changes or improvement in physical activity levels. Studies still in progress or with results unrelated to physical activity were also excluded.

### 3.2. Description of the Included Articles

Six studies were evaluated for inclusion in this review based on the inclusion criteria and removal of articles due to duplication (Table 1). The primary purpose of the six studies included in this review was aimed at identifying exergaming or AVGs as a means of promoting and improving physical activity in adolescents. The ages of the participants in these studies ranged from 12 to 19 years. The study participants, for the most part, were ethnically diverse, with one study composed of only girls. The sample sizes ranged from 20 to 105 participants. The duration of the interventions ranged from 10 weeks to 1 year, with most interventions meeting weekly. None of the studies included in this review used or identified an underpinning framework/theory for the intervention. A variety of games were used, such as Dance Dance Revolution, Camp Conquer, Klub Kinect, and GameBike. All the AVGs encouraged or promoted various levels of physical activity. Data for physical activity measurements were obtained through accelerometers, Fitbits, pedometers, and self-reports (validated questionnaires such as the Perceived Competence Scale). All but one study showed an improvement in physical activity levels and that exergaming or AVGs could actually help slow the decline in moderate-to-vigorous PA over time [13].

## 4. Discussion

Exergames or AVGs appear to be a safe, fun, and valuable means of promoting physical activity in adolescents. The use of exergaming or AVGs could be an innovative alternative for increasing physical activity levels among adolescents. Studies show that playing AVGs over short periods of time are similar in intensity to light-to-moderate traditional physical activities such as walking, skipping, and jogging [16]. It has been established that physical activity declines in adolescents, especially among girls [8,19]. Therefore, effective measures are needed to reduce this trend. The findings in these studies not only showed indications for improved physical activity levels but also improved the overall physical health of adolescents (Table 1).

Although Pope et al. (2018) did not report an improvement or increase in physical activity levels among high school students, they did provide information on how to best structure a gaming intervention for high school students [14]. Adolescents enjoy hanging out with their friends and being interactive. Therefore, lessons learned from this study indicated that separating students from their friends through randomization may not be beneficial. Studies conducted among adolescents indicate that peer support is important for engaging in and maintaining physical activity levels [14,20,21]. Exergaming or AVGs can serve two purposes: promoting physical activity engagement and fostering friendship and social cohesion with their peers, which is an important factor of adolescent socialization. 

The study by Staiano et al. (2017) also revealed the importance of allowing students to tailor their own level of intensity and program choice to reduce boredom and encourage better adherence [15]. Adolescents possess different skillsets when it comes to gaming. Therefore, allowing individuals to progress at their own pace and interest should foster better adherence and compliance. That is what is so unique about exergaming or AVGs—the game can be adjusted or adapted to meet various individuals’ needs and skillsets, and it is not a one-size-fits all concept. 

None of the studies was theory-driven and evidence strongly supports that theory-driven intervention tends to be more effective in changing behavior and thereby achieving desired outcomes [22,23,24]. Health behavior theories (HBTs) should be used when conducting studies related to promoting physical activity [25]. All studies met the criteria of being RCTs, however, small studies such as the one by Ni Mhurchu et al. (#6) [18] could affect RCTs’ reliability.

## 5. Conclusions

The studies in this review show that AVGs are feasible, engaging, and fun. Exergaming or AVGs can be adopted into multiple settings due to their portability and can be implemented in a wide range of environments such as homes, schools, churches, or community centers; and are adaptable to meet various individual skill levels. These findings suggest that, at least in the short term, exergaming or AVGs may be an effective means to increase adolescents’ overall physical activity levels. The main strength of this review is the inclusion of only randomized clinical trials, which considers a higher level of evidence. However, non-randomized studies have also shown that AVGs have the potential to increase movement and energy expenditure in adolescents [21,26,27,28]. This review still leaves us with the question of how best to use exergaming or AVGs to promote and influence engagement in physical activity, and what factors hinder or encourage them to play active games. Studies also need to be conducted that help to establish and adopt standardized protocols regarding the use of exergaming and AVGs in adolescents. We are in the growing age of technology, and the next generation of computer games could play a major role in promoting and encouraging the engagement of physical activity among adolescents.

## Figures and Tables

**Table 1:** Gaming and physical activity (PA): summary of studies that explored the use of active video games (AVGs) or exergames for adolescents.

	Study 1	Study 2	Study 3	Study 4	Study 5	Study 6
Title of Article	Lessons Learned Through the Implementation of an eHealth Physical Activity Gaming Intervention with High School Youth	A Randomized Controlled Trial of Dance Exergaming for Exercise Training in Overweight and Obese Adolescent Girls	Psychological Effects of Dance-Based Group Exergaming in Obese Adolescents	Comparative Effectiveness of a 12-Week Physical Activity Intervention for Overweight and Obese Youth: Exergaming with “Dance Dance Revolution”	Effects of Interactive Video Game Cycling on Overweight and Obese Adolescent Health	Couch Potatoes to Jumping Beans: A Pilot Study of the Effect of Active Video Games on Physical Activity in Children.
Author(s), Year	Pope, L., Garnett, B., & Dibble, M. (2018) [14]	Staiano, A. E, et al. (2017) [15]	Wagener, T. L., Fedele, D. A., Mignogna, M. R., Hester, C. N., & Gillaspy, S. R. (2012) [16]	Maloney, A. E., Threlkeld, K. A., & Cook, W. L. (2012) [13]	Adamo, K. B., Rutherford, J. A., & Goldfield, G. S. (2010) [17]	Ni Mhurchu, C., Maddison, R., Jiang, Y., Jull, A., Prapavessis, H., & Rodgers, A. (2008) [18]
Purpose	To determine the feasibility of incentivizing adolescents to meet physical activity goals through rewards in an electronic game (Camp Conquer).	To determine the feasibility of completing a 12-week supervised, group-based exergaming intervention with overweight and obese adolescent girls self-selecting intensity level and examine the intervention’s effects on body composition and cardiovascular risk factors.	To investigate the impact of dance-based exergaming on a diverse sample of obese adolescents’ perceived competence to exercise, psychological adjustment, and body mass index (BMI) compared with a control condition.	To examine the influence of Dance Dance Revolution (DDR) on total PA among overweight and obese youth. Specifically, to test whether children from the treatment group (DDR and pedometers) would increase their level of PA compared with children from the comparison group (pedometers only) over a 12-week period. The primary outcome was the change in the target child’s level of PA.	To examine the efficacy of interactive video game stationary cycling (GameBike) in comparison with stationary cycling to music on adherence, energy expenditure measures, submaximal aerobic fitness, body composition, and cardiovascular disease risk markers in overweight and obese adolescents, using a randomized controlled trial design.	To evaluate the effect of active video games on children’s physical activity levels.
Framework/Theory	N/A	N/A	N/A	N/A	N/A	N/A
Randomization Process	Randomly assigned to a Game Condition (Camp Conquer) or Control Condition. Control condition received a Fitbit and had access to only an online portal, displaying their daily steps and active minutes.	41 met eligibility criteria and were randomly assigned to either the exergaming intervention (*n* = 22) or control group (*n* = 19).	41 adolescents were randomized: *n* = 21 exergaming; *n* = 20 control. The exergaming condition consisted of a supervised 10-week group dance-based exergame exercise program. The waitlist control condition also spanned 10 weeks in length, and participants were asked to not modify their baseline activity levels during the 10 weeks. Control participants were brought in for consent and baseline and 10-week follow-up assessments but had no other contact with the study team.	*n* = 65: *n* = 32 in the treatment group (DDR+ pedometers); *n* = 33 pedometers only (comparison group). “Dance Dance Revolution.”	30 participants were randomized to video game or music condition: *n* = 15 of the youth were randomly assigned to the experimental group (interactive video game cycling); *n* = 15 youth were randomly assigned to the stationary bike music comparison group.	*n* = 20: *n* = 10 received an active video game upgrade package; *n* = 10 received no intervention (control group).
Dose/Duration	Across 2 years: Year 1 consisted of game development and design; Year 2 consisted of an RCT where the game was tested for efficacy in a group of 100 high school students.	36 hours over 3 months (12 weeks). Each participant in the exergaming condition started the 12-week intervention within 4 weeks of baseline assessment.	10 weeks; returned to the clinic 3 times a week for a 40-min (including two separate 15-min exergaming segments) first session and 75-min (including four 15-min exergaming segments) subsequent sessions.	A 12-week physical activity intervention.	Exercised twice weekly for 10 weeks on the GameBike with the game console turned off.	12-weeks.
Sample/Population	A group of 105 juniors and seniors (high school). Mean age was 17 years with a range from 16 to 18 years of age. 54 out 75 (71%) = female; Hispanic or Latino (12%); Asian (*n* = 9, 12%); African American (*n* = 12, 16%); White (*n* = 50, 67%); Other (*n* = 4, 5%).	41 adolescent girls: 64.3% Black/African American and 35.7% White, with 2.4% of participants reporting Hispanic ethnicity. The average age at enrollment was 16 ± 1.4 years (aged 14 to 18 years).	40 obese adolescents aged 12–18 years (mean age = 14): 66.7% female; 42% (Hispanic); 28% African American/Black; 20% Caucasian/White; and the remaining 10% biracial.	65 families completed the study (32 in the treatment group and 33 control). A child between the ages of 9 and 17 years with a body mass index (BMI) of 85%–94% (overweight category). No indication of race.	30 overweight obese adolescents aged 12–17 years.	20 children mean SD age = 12 ± 1.5 years; 40% female.
Gaming Process/System	Camp Conquer is a capture the flag-style game. The game “currency” was the number of steps students took and the number of minutes they were active each day. Steps and active minutes translated into coins and gems in the game.	The exergaming intervention joined Klub Kinect.	2–3 participants stood on their individual “dance pads” that consisted of colored arrows laid out in a cross shape, and with their feet, participants hit the arrows to musical and visual cues on a screen in front of them.	DDR X with two dance pads to encourage non-solo play (released by Konami Digital Entertainment, Inc.)	Interactive video game cycling program using the GameBike with stationary cycling to music.	Active video game.
Physical Activity Measurement	Each participant was given a Fitbit Flex to track activity throughout the study period. During the 12 experimental weeks, investigators tracked the number of steps students took each day, as well as the number of minutes they were active through data from the students’ Fitbits. Due to Fitbit programming, active minutes were defined as moving for at least 10 minutes continuously at an activity intensity of three metabolic equivalents or more.	Participants reported the amount of time spent exergaming and number of steps taken. Participants used stopwatches and wore Omron GoSmart pedometers.	The Perceived Competence Scale (PCS) (24) measured changes in adolescent-reported competency regarding maintaining regular exercise (e.g., “I feel confident in my ability to exercise regularly”). Target heart rate (THR) goals.	PA levels were tracked for 12 weeks by self-report, pedometer, and accelerometer data.	Adherence to exercise was measured as the number of the times per week participants attended the sessions. Submaximal fitness was assessed using a graded cycle ergometer protocol with the GameBike. Rating of perceived exertion (RPE) was measured and recorded at the end of each 2-min interval using the Borg scale, which ranges in scores from 6 (very low exertion) to 20 (very high exertion—exhausted).	Objective (ActiGraph accelerometer) and subjective (Physical Activity Questionnaire for Children (PAQ-C)) measures. An activity log was used to estimate the time spent playing active and non-active video games.
Results/Findings	When students wore their Fitbits, they did not meet the goal of 10,000 steps/day, nor were they active for 60 min/day. The game group did better with steps and active minutes than the control group, and many students in the game group never played Camp Conquer.	Significant findings were only observed in the subset who adhered to the protocol (i.e., attended at least 75% of the sessions; achieved at least 2600 steps/session), and there were no other significant effects on cardiovascular risk factors. Exergaming reduced body fat and increased bone mineral density (BMD) among those adolescent girls who adhered. Further research is required before exergaming is recommended in clinical settings. The exercise and reduced adiposity involved in this exergaming intervention was sufficient to protect against BMD loss in obese adolescent girls who adhered to the protocol. 36 h of group-based dance exergaming over 12 weeks attenuated adiposity gain and increased BMD among participants who adhered to the intervention, even when adolescents were allowed to self-select intensity levels.	Participants in the dance-based exergaming condition significantly increased in self-reported perceived competence to exercise regularly and reported significant improvement in relation with parents from baseline to end-of-treatment. Dance-based exergaming appears to reduce physical activity barriers by using multiple players, being enjoyable to participants (especially in a group setting), and allowing adolescents to start at an appropriate beginner level, progress to higher levels of difficulty, and develop a sense of competence and mastery.	Self-reported frequency of moderate-to-vigorous PA increased significantly from baseline to 12 weeks for the treatment group and declined for the comparison group. Accelerometer results indicated a significant decline in moderate activity over time for the comparison group. However, there were no significant between-group differences based on the accelerometer or pedometer data.	Both interventions produced significant improvements in submaximal indicators of aerobic fitness as measured by a graded cycle ergometer protocol. Also, when collapsed, the exercise modalities reduced body fat percentage and total cholesterol levels. Significant differences in training intensity were found between groups whereby the average number of minutes per session spent at vigorous intensity (80%-100% of predicted maximum heart rate) was significantly (*p* = 0.05) higher for the music group than the video game group. In addition, the music group exhibited a significantly greater average distance pedaled, measured in kilometers, compared with the video game condition (*p* = 0.03).	Physical activity (counts per minute), measured with an accelerometer, was higher in the active video game intervention group compared to the control group (mean difference at 6 weeks = 194 counts/min (95% confident interval (C.I.) 32, 310), *p* = 0.04; and at 12 weeks = 48 counts/min (95% C.I. −153, 187), *p* = 0.6).
